# Phylogeography, Interaction Patterns and the Evolution of Host Choice in *Drosophila*-Parasitoid Systems in Ryukyu Archipelago and Taiwan

**DOI:** 10.1371/journal.pone.0129132

**Published:** 2015-06-12

**Authors:** Biljana Novković, Masahito T. Kimura

**Affiliations:** Graduate School of Environmental Science, Hokkaido University, Sapporo, Hokkaido, Japan; University of Innsbruck, AUSTRIA

## Abstract

Island biotas provide a great opportunity to study not only the phylogeographic patterns of a group of species, but also to explore the differentiation in their coevolutionary interactions. *Drosophila* and their parasitoids are exemplary systems for studying complex interaction patterns. However, there is a lack of studies combining interaction-based and molecular marker-based methods. We applied an integrated approach combining phylogeography, interaction, and host-choice behavior studies, with the aim to understand how coevolutionary interactions evolve in *Drosophila*-parasitoid island populations. The study focused on the three most abundant *Drosophila* species in Ryukyu archipelago and Taiwan: *D*. *albomicans*, *D*. *bipectinata*, and *D*. *takahashii*, and the *Drosophila*-parasitoid *Leptopilina ryukyuensis*. We determined mitochondrial COI haplotypes for samples representing five island populations of *Drosophila* and four island populations of *L*. *ryukyuensis*. We additionally sequenced parts of the autosomal *Gpdh* for *Drosophila* samples, and the ITS2 for parasitoid samples. Phylogenetic and coalescent analyses were used to test for demographic events and to place them in a temporal framework. Geographical differences in *Drosophila*-parasitoid interactions were studied in host-acceptance, host-suitability, and host-choice experiments. All four species showed species-specific phylogeographic patterns. A general trend of the haplotype diversity increasing towards the south was observed. *D*. *albomicans* showed very high COI haplotype diversity, and had the most phylogeographically structured populations, with differentiation into the northern and the southern population-group, divided by the Kerama gap. Differentiation in host suitability was observed only between highly structured populations of *D*. *albomicans*, possibly facilitated by restricted gene flow. Differentiation in host-acceptance in *D*. *takahashii*, and host-acceptance and host-choice in *L*. *ryukyuensis* was found, despite there being no differentiation in these two species according to molecular markers. Host choice assays show that *L*. *ryukyuensis* populations that have had more time to coevolve adapt their behavior to exploit the most suitable host – *D*. *albomicans*. *L*. *ryukyuensis* parasitoids on border ranges may, on the other hand, benefit from broader host-acceptance, that may facilitate adaptation to uncertain and variable environments. All results indicate that *Drosophila*-parasitoid populations in the Ryukyu archipelago and Taiwan have different evolutionary trajectories, and coevolve in a dynamic, complex, and local-specific way.

## Introduction

In evolutionary and ecological studies attention is increasingly shifting to interspecific interactions and coevolutionary systems. Coevolution is one of the driving forces in the organization of biodiversity, and the last two decades have seen much research directed at unraveling complex interaction webs and their dynamic patterns [1–3]. Host-parasitoid systems, especially *Drosophila* and their parasitoids, are exemplary coevolutionary study-systems due to the fact that their interactions can be studied both in the field and the laboratory, and their short generation times allow us to observe evolutionary phenomena over relatively short time-scales.

Over the years, host-parasitoid interaction studies have focused on comparative phylogeography [4–7], or more often interaction traits [8–10], but these approaches have rarely been combined [11]. Additionally, no comparative phylogeographic studies of *Drosophila* and their parasitoids have been conducted so far. Here we use an integrated approach, studying phylogeography, interaction, and host choice behavior in *Drosophila-*parasitoid systems, with an aim to deepen the understanding of how these interactions evolve in more or less isolated populations.

In this paper we focus on the geographic area of the Ryukyu archipelago and Taiwan. Encompassing about 150 subtropical islands and stretching 1,300 km in the northeast-southwest direction, this area has heterogeneous flora and fauna associated with land bridge formation and submersion due to glaciation associated sea level changes [12, 13]. Two deep straits in this island system, the Tokara strait and the Kerama strait, are two barriers that have shaped genetic boundaries for many species of this region [14–16]. Considering that isolated populations are more likely to follow different evolutionary routes, island biotas provide a great opportunity to study not only the speciation and phylogeographic patterns of a group of species, but also to explore the differentiation in their coevolutionary interactions.

Our study species are the three most common fruit-feeding *Drosophila* species of the Ryukyus: *D*. *albomicans* (Duda, 1924), *D*. *bipectinata* Duda, 1923 and *D*. *takahashii* Sturtevant, 1927, and a common *Drosophila* larval parasitoid *Leptopilina ryukyuensis* Novković and Kimura, 2011. Traditionally, parasitoids are studied only in relation to their current hosts, and in this study that would be *D*. *albomicans*. Nonetheless, we decided to include two abundant species that this parasitoid is most likely to interact with in the wild. Selection should favor parasitoids that can exploit the most abundant host species [17, 18]. However, this selective pressure may vary on spatial and temporal scales due to differences in local community structure and dynamics, favoring host-shifts, and different evolutionary trajectories in different populations [19]. By including abundant species other than the current host, we aim to find hints of past interactions, and shed more light on interaction potential and host-choice behavior.

Using samples from five island populations of flies, four island populations of the parasitoid, and three laboratory strains for each study species we conducted the following analyses: (1) We used mitochondrial COI partial sequences, and the partial sequences of the autosomal *Gpdh* for flies, and ITS2 for wasps, to explore the phylogeography of the four insect species and determine to what extent the phylogeography of the three *Drosophila* and the parasitoid *L*. *ryukyuensis* in this area are shaped by barriers to gene flow, and/or coevolutionary interactions; (2) Next, we tested for differentiation in host-parasitoid interactions by host-acceptance and host-suitability experiments in laboratory; (3) Finally, we explored the host choice of *L*. *ryukyuensis* in a two-choice assay, designed based on the outcomes of host-acceptance and host-suitability experiments. Our results indicate that *Drosophila*-parasitoid island populations have different evolutionary trajectories, and coevolve in a dynamic, complex and local-specific way.

## Materials and Methods

### Study species

**Fig 1:**
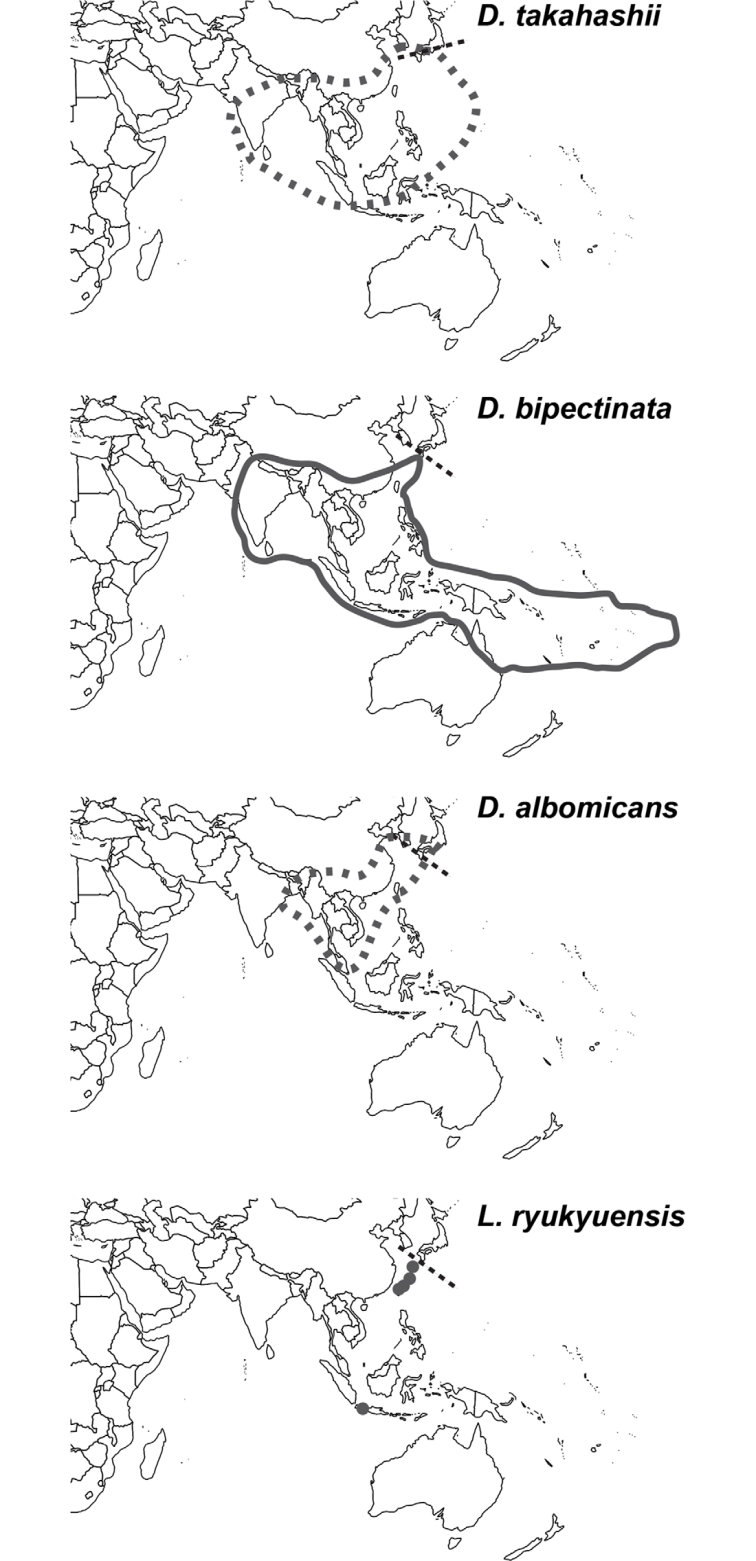
Estimates of distribution for the four study species. Distribution maps were based on available previous studies [23, 25–28]. Further studies are required to establish the exact distribution of *L*. *ryukyuensis*, and confirm distributions of *D*. *takahashii* and *D*. *albomicans* in regions where disambiguation from sister-species has proved challenging. Dotted lines indicate the northernmost border of the natural distribution for these four species.

*D*. *albomicans*, *D*. *bipectinata*, and *D*. *takahashii* are the most common frugivorous *Drosophila* in the Ryukyu archipelago. Their estimated distributions are shown in Fig 1. In previous studies carried out in this area, both *D*. *takahashii* and *D*. *bipectinata* were sampled mainly from open lands and domestic areas, while *D*. *albomicans* was mainly collected from the forest [20–22]. *L*. *ryukyuensis* is a koinobiont hymenopteran larval parasitoid with reported samplings from Ryukyu islands, Taiwan, and Indonesia [23, 24]. In surveys in southern Ryukyus, this species was, so far, recovered mainly from *D*. *albomicans* pupae, and occasionally from *D*. *lacteicornis*, *D*. *quadrilineata*, and *D*. *daruma* [22].

### Sample collection and strains

**Fig 2:**
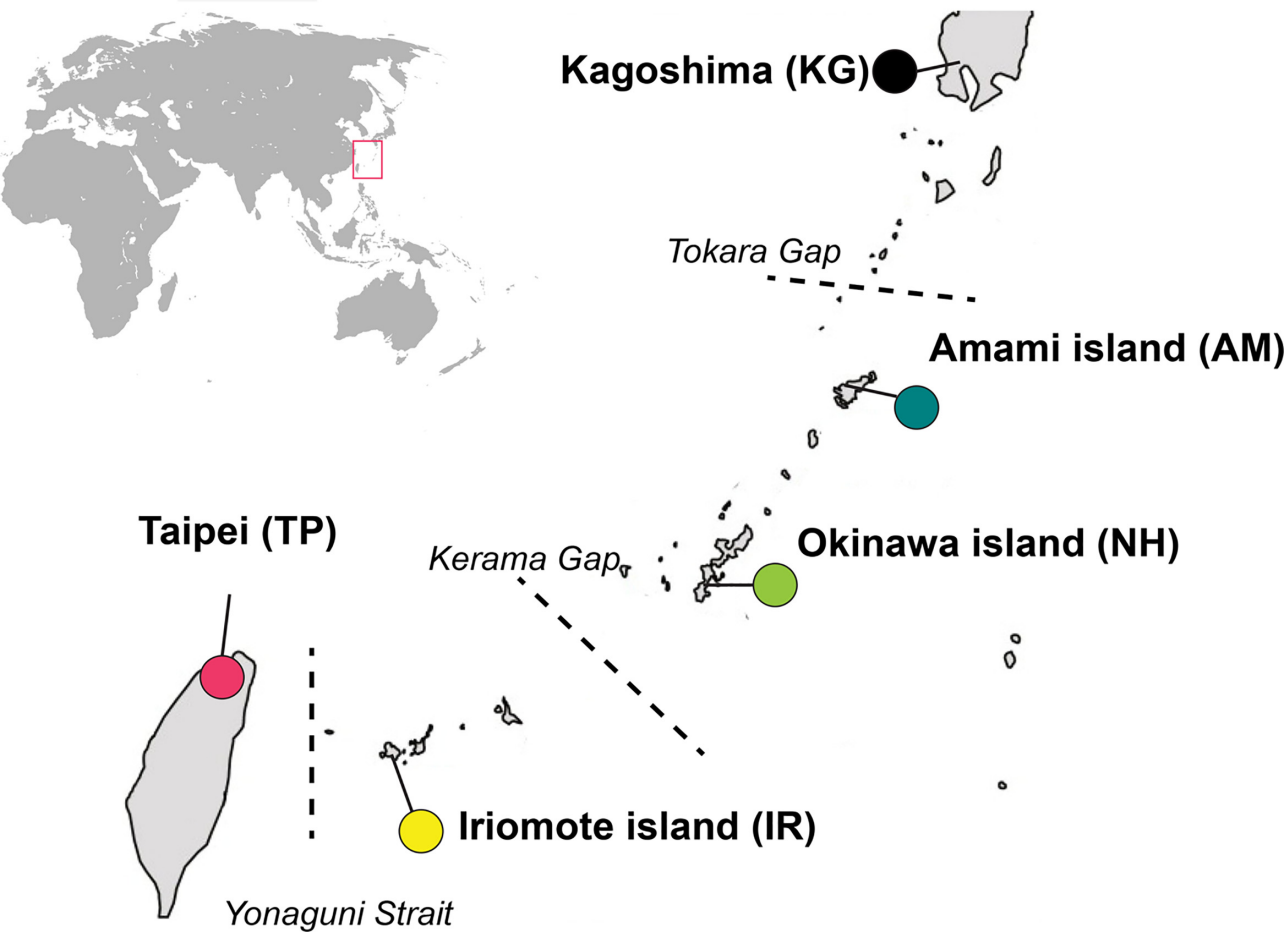
Map of the Ryukyu archipelago and Taiwan with the position of the collection sites. Colored circles denote collection sites. Location abbreviations are given in the brackets. Tokara, Kerama, and Yonaguni straits are roughly indicated by dashed lines.

Samples were collected from five localities: Kagoshima in Kyushu island, southern Japan (KG: 35,640 km^2^, October 2009; 31.60 N, 130.55 E), Amami island (AM: 712 km^2^, October 2009; 28.37 N, 129.49 E), Okinawa island (NH: 1,201 km^2^, October 2009; 26.23 N, 127.71 E) and Iriomote island (IR: 289 km^2^, October 2009; 24.39 N, 123.84 E) in the Ryukyu archipelago, and Taipei in Taiwan (TP: 35,883 km^2^, June 2010; 25.03 N, 121.60 E) (Fig 2). No specific permission was required for these field studies, because our study did not involve endangered or protected species. Traps containing banana were placed in the field, seven traps per location. Each trap was set in a different microhabitat, and/or at a different altitude. Samples for the molecular analyses were collected 5–7 days later, directly at each location, and consisted of flies and wasps that were attracted to the baits. To establish laboratory strains, traps were brought back to the laboratory. When host (i.e., *Drosophila*) pupae were formed in the containers, they were placed in Petri dishes without identification, and then examined for the emergence of flies and wasps. Each strain was established from multiple traps and was kept together as a population. Laboratory strains were successfully established from Amami, Okinawa and Iriomote islands, but the AM *D*. *albomicans* strain was lost after a couple of generations. Strains of *D*. *bipectinata* and *D*. *albomicans* were additionally obtained from Taipei. *Drosophila* strains were reared on corn-malt medium. Wasp strains were reared on *D*. *simulans* Sturtevant, 1919 as host. *D*. *simulans* is not native to East Asia and was chosen in order to avoid the effect of adaptation. In our experience, this species is non-resistant to a number of *Leptopilina* parasitoids including *L*. *ryukyuensis*, and rearing on *D*. *simulans* for several years has not caused discernable changes to wasp counter-defense towards other host species. Rearing and all subsequent experiments were conducted at a constant temperature of 23°C under a 15 h light—9 h dark condition. Experiments were performed 6–30 generations after strain collection.

### Molecular techniques

We extracted genomic DNA from 255 fly and 36 wasp specimens following a modified phenol-chloroform protocol. All amplifications were performed in 25 μl reaction volumes using *AmpliTaq Gold* DNA polymerase (Invitrogen Corporation Carlsbad, CA, USA), with primer annealing at 50°C. We amplified a 574–612 bp fragment of the mitochondrial gene for cytochrome c oxidase subunit I (COI) for all four species, a 340–431 bp fragment of the autosomal gene for glycerol-3-phosphate dehydrogenase (*Gpdh*) for the three fly species, and a 586 bp fragment of the intertranscribed spacer sequence II of ribosomal RNA genes (ITS2) for the wasp. The amplified *Gpdh* fragment includes parts of exon 3 and 4, and the complete intron 3. *Gpdh* and ITS2 amplifications were not successful in 20 fly and 14 wasp samples. DNA extraction protocol, PCR protocol and the combinations of primers used for the amplification are given in S1 Supporting Information. PCR products were sequenced with the same primers used in the PCR protocol, using Big Dye Terminator Cycle Sequencing Kit (ABI). Sequencing was carried out with a 3100 Genetic Analyzer (Applied Biosystems, Foster City CA, USA). *Gpdh* sequences with more than one polymorphic site were re-sequenced utilising primers designed according to divergent nucleotide sites (S1 Supporting Information). Sequences obtained by different primers were assembled by ProSeq v3.2 [29]. *Gpdh* sequences that could not be resolved by direct sequencing were amplified using PrimeSTAR GXL DNA Polymerase kit (Takara Mirus Bio, Madison, WI, USA) following the manufacturer`s protocol, blunt-end cloned using competent high DH5α cells (Toyobo DNA-903) according to the manufacturer’s protocol, and then re-sequenced (accession numbers: KP863175—KP863465, KR056305—KR056818).

### Host acceptance and suitability

Host acceptance experiments are no-choice assays that are important to ascertain the innate potential of parasitoids to parasitize a certain host, an ability that could otherwise be masked in choice assays by a preference for higher-ranked hosts [30]. To determine host acceptance for parasitoid strains, second instar drosophilid larvae (up to 80) were placed in a Petri dish (3 cm in diameter) containing a small amount of cornmeal-malt medium. Five 5–10 days old wasp females experienced on patches of *D*. *simulans* were introduced, and left to oviposit for four hours. Following the removal of wasps, we dissected the larvae and checked for the presence of wasp eggs. Experiments were performed for all wasp and fly strains reciprocally. The oviposition rate was calculated as the number of parasitized larvae per total number of larvae (experiment data are provided in S1 Table).

Host suitability experiments indicate the relative differences in host defense and parasitoid counter-defense between species and populations. To estimate host suitability of flies for wasp strains from different localities, we placed two-day-old fly larvae in a Petri dish containing a small amount of medium, and exposed them to five 5–10 days old wasp females experienced on patches of *D*. *simulans* for 24 hours. Fly larvae were then transferred into vials with medium. Additional larvae were dissected from each dish to confirm oviposition. The vials were regularly checked for the emergence of flies and/or wasps. *Drosophila* strains with higher wasp emergence and lower fly emergence were considered more suitable as hosts (experiment data are provided in S2 Table).

### Host Choice Experiments

We focused on the host-choice of AM and IR *L*. *ryukyuensis* strains based on the results of host-acceptance and host-suitability experiments. These wasp strains were given a choice between *D*. *albomicans* and *D*. *bipectinata*, both NH strains. In this way we were able compare how wasps respond to the exact same *Drosophila* strains, without the effect of local adaptation. However, to confirm that the wasps react in the same way to allopatric and sympatric host strains of these two species, we preformed additional experiments with the IR wasp strain and IR fly strains.

At the beginning of every experiment, 30 second-instar larvae of *D*. *albomicans* and *D*. *bipectianta* each were added to a Petri dish containing a small amount of corn-malt medium. To differentiate between them, one of the species was reared on a medium containing red food dye (carmin), coloring the digestive tract of the larvae. To exclude the possible effect of color on host choice, both *D*. *albomicans* and *D*. *bipectinata* were alternatively colored in half of the experiments. A single wasp was inserted into the thus prepared Petri dish and monitored for oviposition. We recorded the sequence of the first 20 successful oviposition events, and the rejection events between ovipositions. Successful oviposition was determined by characteristic ovipositor movements, and an ovipositor insertion longer than 10 s. We recorded rejection when the ovipositor was removed in less than 5 s. We suspect that in some cases wasps make decisions in a very brief time frame (<1 s), but in these cases it was hard to judge if the ovipositor was inserted or not, and these events were not included in the total rejection count. In other words, the recorded number of rejected larvae is likely an underestimate of the real number of rejections. After oviposition in a specific larva was confirmed, that larva was immediately removed from the Petri dish. Additional larvae of the same species were then added in order to maintain the same larval density and encounter probability. Larvae that were oviposited in were later dissected to confirm the presence of eggs. The sequence of 20 oviposition events was observed for 15 wasp individuals for each color/*Drosophila* strain/wasp strain combination (90 wasps and 1800 oviposition events observed in total).

### Data analyses

Haplotype variation within and among populations was assessed using DAMBE 5.2 [31]. Arlequin 3.5 [32] was used to estimate haplotype diversity (Hd) and nucleotide diversity (π) indices, and pairwise population genetic distances. Best-fit substitution models were selected based on the Bayesian Information Criterion in MEGA 6 [33]: the T92+G model for *Drosophila* COI, the JC model for *Drosophila Gpdh*, and the T92 model for COI and ITS2 of *L*. *rukyuensis*. Phylogeny was assessed by Bayesian inference (BI) as implemented in MrBayes 3.2.1 [34]. We employed two substitution types (“nst = 2”) with rate variation across sites modeled using a gamma distribution (“rates = gamma”) for *Drosophila* COI, one substitution type (“nst = 1”) with equal stationary state frequencies (“statefreqpr = fixed(equal)”) for *Drosophila Gpdh*, and two substitution types (“nst = 2”) for *L*. *ryukyuensis* COI and ITS2. Default parameters for the Metropolis-coupled Markov chain Monte Carlo (MCMCMC) were used. Two separate runs were processed simultaneously (three hot chains and one cold chain each), running each partitioning scheme for one million generations. The mean for the unconstrained exponential prior on branch lengths was set to 0.01. Trees were sampled every 1000 generations. First 25% were discarded as burn-in. Tree topology and branch length were based on the 50% majority-rule consensus tree and its associated posterior probabilities. Much of the structure in resulting BI phylogeny was shallow, so we proceeded to construct median-joining networks of COI, *Gpdh*, and ITS2 haplotypes using NETWORK 4.6 (fluxus-engineering.com) [35]. Differences between populations were tested with analysis of molecular variance AMOVA in Arlequin [36]. Tajima`s *D* statistics [37] and Fu`s *Fs* [38] were used to detect deviations from the pattern of polymorphism expected from a neutral evolution model. Demographic and spatial expansion models were calculated in Arlequin and fitted with the data (1000 permutations). To test the validity of the sudden expansion model, SSD, the sum of square deviations between the observed and expected mismatch, was used [39]. Genetic distances were expressed through Slatkin`s linearized *F*_ST_ [40], and the matrices of genetic distances and geographical distances were compared using Mantel`s test [41] with 1000 permutations in Arlequin. Unit of mutational time τ for COI was used to determine the time elapsed since the expansion events for *D*. *albomicans* and *D*. *takahashii*, based on a mutation rate (μ) of 5.88×10^–7^ mutations per sequence per generation [42, 43], at 10 generations per year. Expansion time was not estimated for *D*. *bipectinata* and *L*. *ryukyuensis* due to the low resolution of COI haplotypes.

Host acceptance and suitability were analyzed using generalized linear models (GLMs) with binomial error and logistic (logit) link function. Each data-set was tested twice, once with fly strains and once with wasp strains set as predictor variables. In host acceptance experiments, the response variable was the percentage of larvae oviposited in. In host suitability experiments the proportion of flies versus wasps eclosed was set as the response variable. Significant differences between the strains in a data-set were tested by Chi-square (χ^2^) tests. To check for differences between pairs of populations we employed a Fisher`s exact probability test, followed by Holm`s method for multiple comparisons. Binomial test was used to determine whether the host choice of the subset of interest differed significantly from the expected random-choice hypothesis. Host choice between subsets was compared using a 2-sample test for equality of proportions without continuity correction. All analyses were carried out in R statistical software version 2.13.0 [44].

## Results

### Phylogenetics, population structure and population history

Sample size, number of haplotypes, number of polymorphic sites per population, haplotype diversity, and nucleotide diversity are given in Table 1. COI showed more diversity than *Gpdh* in *D*. *takahashii* and *D*. *albomicans*, while *Gpdh/*ITS2 was more diverse compared to COI in *D*. *bipectinata* and *L*. *ryukyuensis*. We observed a trend where the haplotype and nucleotide diversity increased towards the south. This pattern was especially pronounced in the COI sequences of *D*. *takahashii* and *D*. *albomicans*. All *Gpdh* substitutions were either synonymous or located in the noncoding region. One out of 30 and one out of 58 substitutions were non-synonymous for the COI sequences of *D*. *takahashii* and *D*. *albomicans*, respectively.

**Table 1:** Sample sizes and summary of the DNA polymorphism measures for *Drosophila takahashii*, *D*. *bipectinata*, *D*. *albomicans*, and *Leptopilina ryukyuensis* in the Ryukyu archipelago and Taiwan.

	COI						*Gpdh*						ITS2					
*Population*	N	H	P	Hd		π (%)	N	H	P	Hd		π (%)	N	H	P	Hd		π (%)
***D*. *takahashii***																		
KG	17	5	6	0.691	±0.075	1.103	18	2	1	0.056	±0.052	0.056						
AM	12	5	4	0.803	±0.078	1.227	11	1	0	0	±0.000	0						
NH	17	8	10	0.838	±0.068	2.176	17	2	1	0.337	±0.083	0.337						
IR	17	11	13	0.926	±0.045	2.853	17	2	1	0.059	±0.055	0.059						
TP	21	13	24	0.890	±0.060	3.009	18	1	0	0	±0.000	0						
*Total*	*84*	*32*	*30*	*0*.*911*	±*0*.*021*	*2*.*45*	*81*	*2*	*1*	*0*.*106*	±*0*.*032*	*0*.*11*						
***D*.*bipectinata***																		
KG	4	1	0	0	±0.000	0	4	4	3	0.75	±0.139	0.929						
AM	16	1	0	0	±0.000	0	16	7	5	0.853	±0.028	1.458						
NH	20	1	0	0	±0.000	0	19	9	8	0.836	±0.036	1.582						
IR	17	2	1	0.382	±0.113	0.382	16	9	7	0.857	±0.035	1.718						
TP	13	2	1	0.154	±0.126	0.154	12	5	4	0.746	±0.052	1.004						
*Total*	*70*	*3*	*2*	*0*.*136*	±*0*.*054*	*0*.*14*	*67*	*10*	*8*	*0*.*838*	±*0*.*014*	*1*.*47*						
***D*. *albomicans***																		
KG	2	2	3	1	±0.500	3	2	3	5	0.833	±0.222	3.33						
AM	24	8	16	0.659	±0.106	2.424	20	3	5	0.580	±0.049	2.164						
NH	24	8	11	0.696	±0.095	1.283	20	3	4	0.542	±0.056	1.892						
IR	24	20	35	0.982	±0.018	6.743	24	4	6	0.584	±0.037	2.083						
TP	27	19	27	0.966	±0.020	7.100	21	6	6	0.77	±0.038	1.733						
*Total*	*101*	*48*	*58*	*0*.*916*	±*0*.*023*	*5*.*85*	*87*	*7*	*7*	*0*.*644*	±*0*.*026*	*2*.*05*						
***L*. *ryukyuensis***																		
AM	10	1	/	/	/	/							10	1	0	0	±0.000	0
NH	6	1	/	/	/	/							3	1	0	0	±0.000	0
IR	18	1	/	/	/	/							8	4	2	0.650	±0.1051	0.900
TP	2	1	/	/	/	/							1	2	1	1.000	±0.500	1.000
*Total*	*36*	*1*	/	/	/	/							*22*	*4*	*2*	*0*.*3584*	±*0*.*087*	*0*.*510*

(N) sample size, no. of haplotypes (H), no. of polymorphic loci (P), haplotype diversity (Hd) and nucleotide diversity (π) for COI, *Gpdh* and ITS2 are shown.

**Fig 3:**
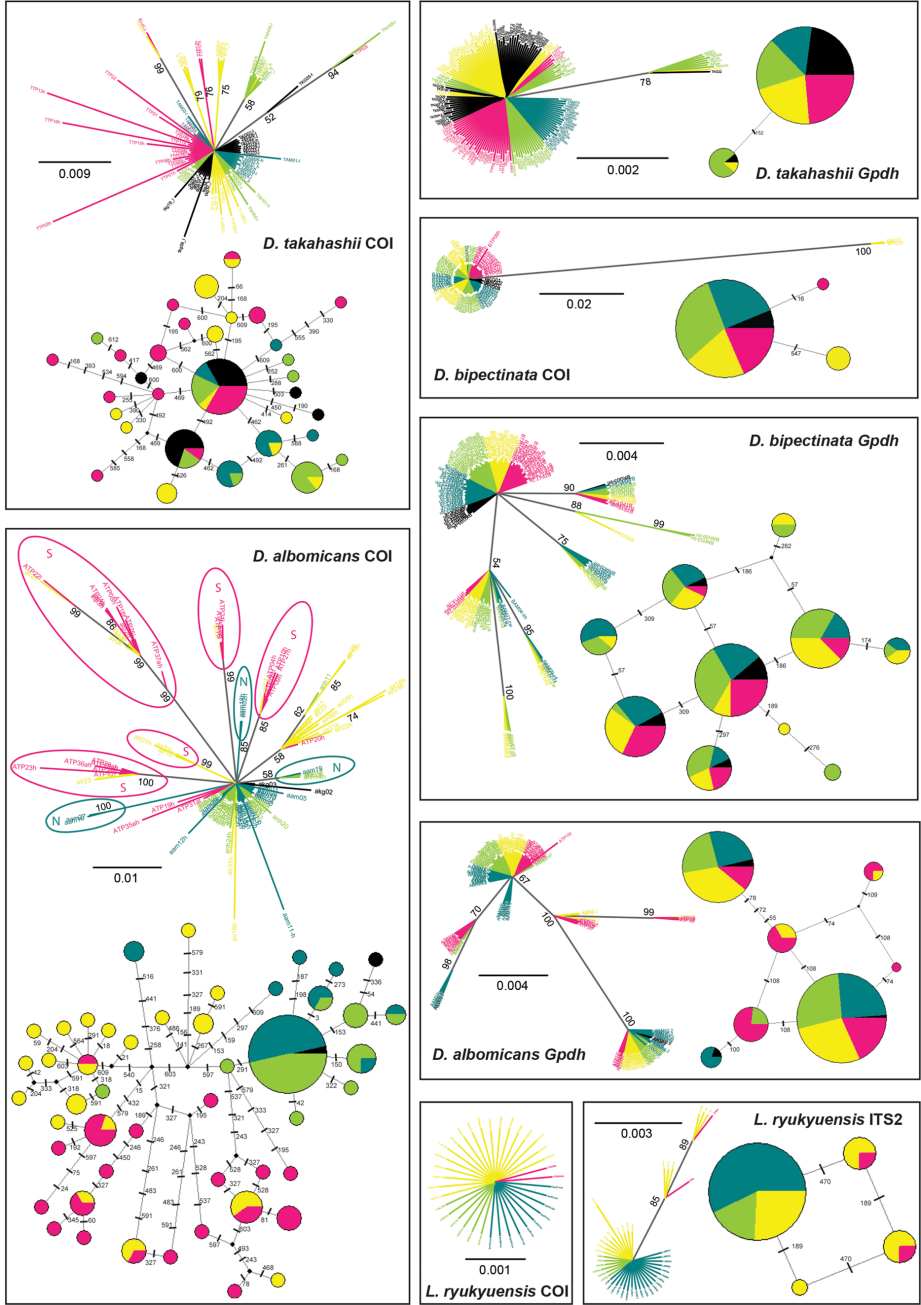
Unrooted consensus trees estimated with Bayesian inference and MJ networks. Trees and networks for *D*. *takahashii* COI (612 bp) and *Gpdh* (431 bp), *D*. *bipectinata* COI (574 bp) and *Gpdh* (340 bp), *D*. *albomicans* COI (611 bp) and *Gpdh* (401 bp), and *L*. *ryukyuensis* COI (649 bp) and ITS2 (586 bp) are given. Colors represent different populations: KG (black), AM (blue), NH (green), IR (yellow), and TP (red). Posterior probabilities of 0.5 or greater are shown.

**Fig 4:**
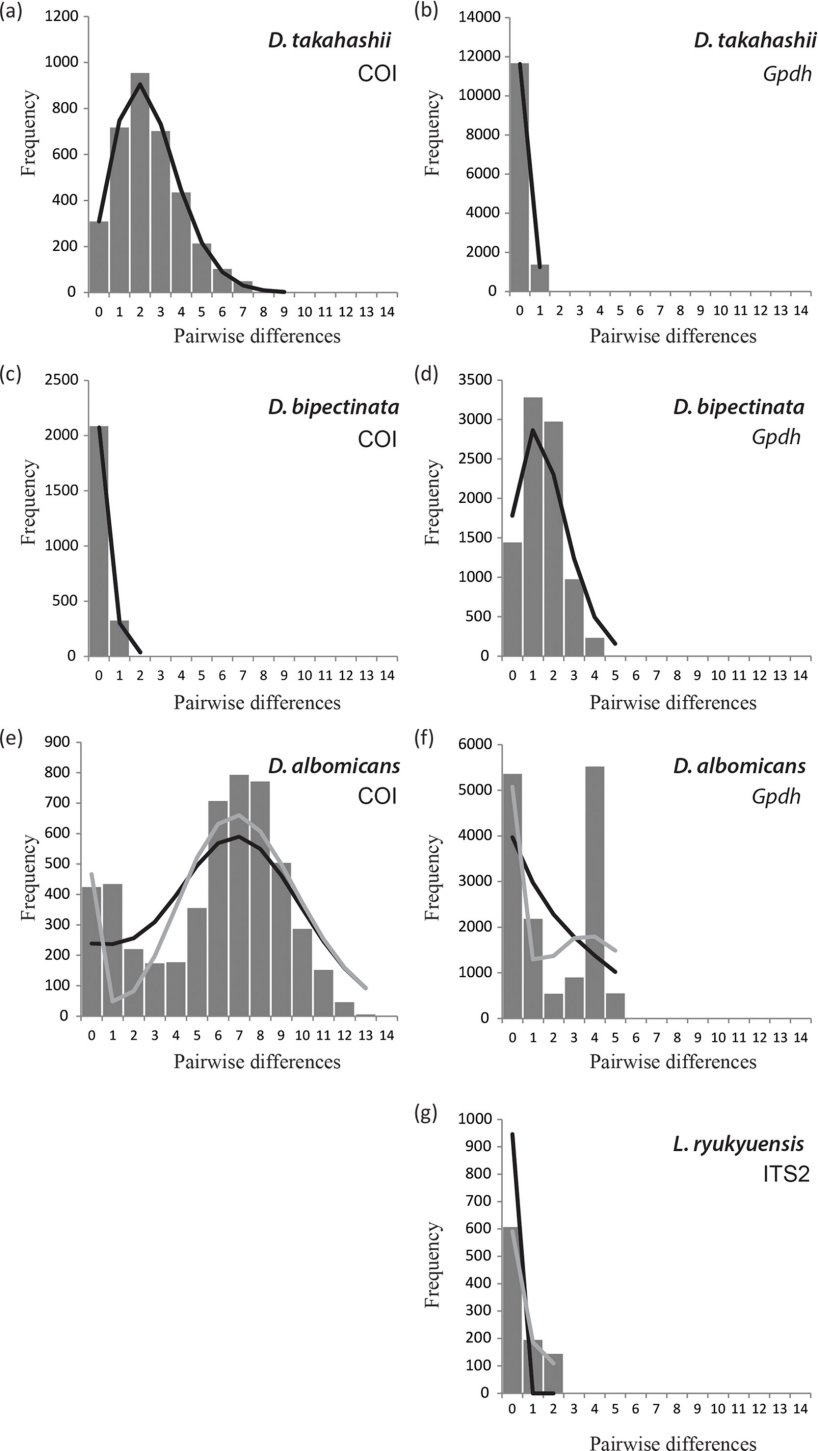
Pairwise mismatch distribution calculated for mitochondrial and nuclear markers of the four study species. Solid histograms represent observed differences, black line the expected distribution compatible with a sudden-expansion model, and gray line the distribution compatible with the spatial expansion model. Where both models overlap, only the black line is shown.

Unrooted majority-rule consensus trees and more detailed median-joining networks of the four species are shown in Fig 3. Geographic structure with strongly supported subclades was observed only in the COI tree of *D*. *albomicans*. No similar geographical pattern was observed neither in the *Gpdh* of this species, nor in any of the trees of *D*. *takahashii*, *D*. *bipectinata*, or *L*. *ryukyuensis*. The following observations can be drawn from both the phylogenetic and network analyses:

1. *D*. *takahashii* COI haplotype network exhibited a star-like pattern, with one core haplotype shared by all five populations. The number of haplotypes ranged from five in KG and AM, to 13 in TP. Haplotype and nucleotide diversity were highest in the two southern populations (IR, TP). While some highly supported clades belonged to single populations, the majority was shared. *Gpdh* had only two haplotypes.
2. *D*. *bipectinata*`s haplotype diversity was much lower for COI compared to *Gpdh*. There was no discernable geographic differentiation. Four out of 10 *Gpdh* haplotypes were distributed in all sampled populations.
3. *D*. *albomicans* had the highest nucleotide diversity for both COI and *Gpdh*. COI haplotype network was complex and highly diversified, and the total COI nucleotide diversity (π) was very high at 5.85%. The number of COI haplotypes within each *D*. *albomicans* population ranged from two for KG, to 20 for IR. Three highly supported clades were formed exclusively by samples belonging to the northern (KG, AM, NH), and five by samples belong to the southern populations (IR, TP). We found no shared haplotypes for the northern and southern population groups. The southern populations were much more diverse with average uncorrected p-distances of 1.1 and 1.2% within the IR and TP populations. There was considerable differentiation between the northern (KG, AM, NH) and the southern (IR, TP) populations based on the *F*_ST_ value (*F*_ST_ = 0.34438, *P*<0.0001). High diversity representing 63.2% of the total variation was observed within populations (AMOVA; *P*<0.0001), while the variation between the northern and southern populations represented 32.3% of the total variation within the species (AMOVA; *P* = 0.097). Average uncorrected p-distances between northern and southern populations ranged from 0.4 to 0.5%. On the other hand, no differentiation was observed in *Gpdh*, where the two most common haplotypes were shared by all five populations.
4. *L*. *ryukyuensis* showed a similar pattern to *D*. *bipectinata*, as far as the low mitochondrial diversity is concerned, with a single haplotype shared by all studied specimens. Four ITS2 haplotypes were found in this species, with the highest diversity in IR. To gain more insight into the demographic history of these species, we employed mismatch distribution analyses, based on the pairwise differences among individuals in our meta-populations. In populations that have been in equilibrium over a long period of time, these distributions become ragged and erratic, while populations that have passed through recent demographic expansion have a smooth one-peak mismatch distribution [45]. A unimodal mismatch distribution was obtained for COI and *Gpdh* sequences of *D*. *takahashii*, *D*. *bipectinata*, and *L*. *ryukyuensis* (Fig 4), indicating recent expansion or selection. *D*. *albomicans* had bimodal COI and *Gpdh* distributions. The first peak of COI in this species roughly corresponds to the expansion event of the northern populations, and the second to the expansion event of the southern populations (S1 Fig). SSD values were low and non-significant for COI and *Gpdh* of *D*. *takahashii*, and COI of *D*. *bipectinata* and *D*. *albomicans*, for both demographic and spatial expansion mismatch models, indicating the data are a good fit for both models (Table 2). *Gpdh* of *D*. *bipectinata* was not a good fit for either model, while *L*. *ryukyuensis Gpdh* data fitted the spatial but not the demographic expansion model. Significant negative Tajima`s *D* values and highly significant negative values of Fu`s *Fs* for COI, indicating an excess of recent mutations, support the occurrence of population range expansion for *D*. *albomicans* and *D*. *takahashii* (Table 2). The population expansion event of *D*. *takahashii* was estimated to have occurred c. 196 ka (151–255 ka). The first expansion event of *D*. *albomicans* was estimated at c. 724 ka (497–866 ka) and the second at c. 122 ka (15–242 ka). No significant relationship between genetic divergence and geographic distance was observed for any of the species (Mantel test, 1000 permutations) (S2 Fig).

**Table 2:** Summary of historical demographic expansion analyses for *Drosophila takahashii*, *D*. *bipectinata*, *D*. *albomicans*, and *L*. *ryukyuensis*.

	*D*—test	*Fs*—test	SSD (*P*-value)	τ (95%CI)
***D*. *takahashii***				
COI	-1.83817**	-26.74479***	0.00040 (0.79)	2.31 (1.77–3)
*Gpdh*	-0.42144	-0.22013	0.00008 (0.3)	3.00 (0.29–3.00)
***D*. *bipectinata***				
COI	-1.10330	-1.89220	0.00044 (0.3)	3.00 (0.58–3)
*Gpdh*	0.01688	-1.6938	0.01123 (0.00)	1.61 (1.35–1.89)
***D*. *albomicans***				
COI	-1.52756*	-25.21435***	0.01202 (0.18)	7.80 (3.94–10.35)62)
*Gpdh*	1.46274	2.06776	0.10466 (0.1)	5.04 (0.00–6.78)
***L*. *ryukyuensis***				
COI	/	/	/	/
ITS2	0.20351	-0.87232	0.19408 (0.00)	0.00 (0.00–0.74)

*D*-test, Tajima`s *D*-test [35]; *Fs*-test, Fu`s *Fs*-test [36]; SSD, sum of squared deviations between the observed [38], and the expected mismatch and τ, scaled time elapsed since the demographic event; are shown.

*, *P*<0.05;

**, *P*<0.01;

***, *P*<0.001.

### Host acceptance and suitability

**Fig 5:**
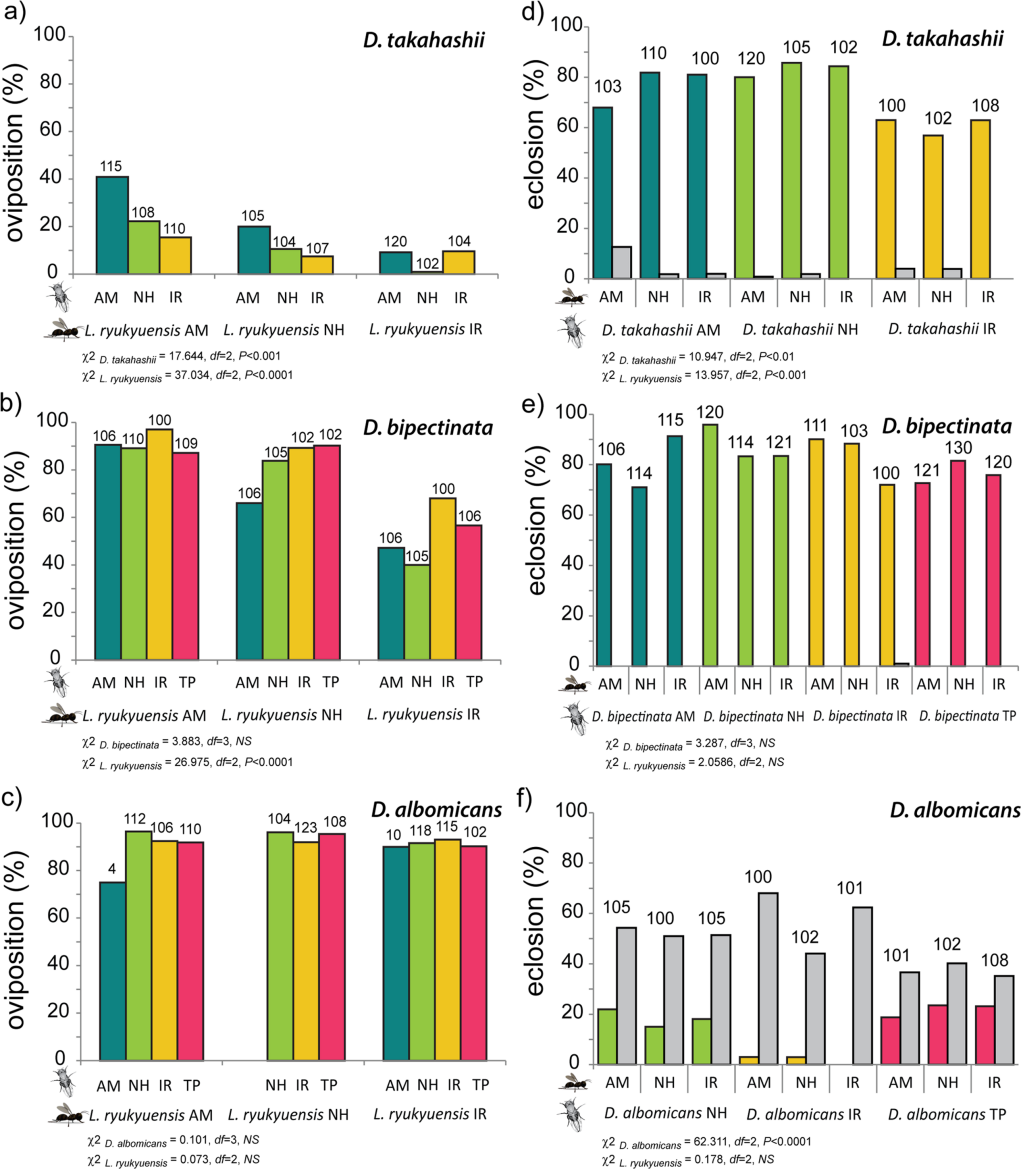
Host acceptance and host suitability. Left column shows the host acceptance of AM, NH, and IR strains of *L*. *ryukyuensis* for each strain of *D*. *takahashii*, *D*. *bipectinata*, and *D*. *albomicans*. Right column shows the emergence of flies (in colour) and wasps (gray) in host suitability experiments. Numbers above each bar indicate the number of host larvae tested. Colours represent different fly populations: AM (blue), NH (green), IR (yellow), and TP (red). Chi-squared and *P* values obtained for fly strains as predictor variables, and wasp strains as predictor variables, are given below each graph.

*L*. *ryukyuensis* readily oviposited in *D*. *bipectinata* and *D*. *albomicans* larvae, accepting them as hosts, but was much more reluctant towards the larvae of *D*. *takahashii* (Fig 5). All wasp strains equally accepted *D*. *albomicans* larvae, but significant differences were observed when they oviposited in *D*. *takahashii* and *D*. *bipectinata* (Chi-squared: χ^2^ = 37.034, *df* = 2, *P*<0.0001; χ^2^ = 26.975, *df* = 2, *P*<0.0001). The AM wasp strain more readily accepted *D*. *takahashii* (Fisher’s: AM-NH: *P*<0.001, AM-IR: *P*<0.0001), while the IR wasp strain was least likely to oviposit in this fly species (Fisher’s: IR-AM: *P*<0.0001, IR-NH: *P*<0.05). IR wasps were additionally more reluctant to oviposit in *D*. *bipectinata* larvae (Fisher’s: IR-AM: *P*<0.0001, IR-NH: *P*<0.001). Differences between fly strains were observed only in *D*. *takahashii* (Chi-squared: χ^2^ = 17.644, *df* = 2, *P*<0.001), where the AM strain was significantly more readily accepted as a host compared to the other two fly strains (Fisher’s: AM-NH, AM-IR: *P*<0.01).

Based on host suitability experiments, *D*. *takahashii* was not a good host for *L*. *ryukyuensis*, with wasp emergence under 12.6% in all tested combinations (Fig 5). *D*. *bipectinata* was the least suitable host with under 1% wasp emergence. *D*. *albomicans* was the most suitable host for *L*. *ryukyuensis*, with wasp emergence of up to 68%. Significant differences between the strains were found for this species (Chi-squared: χ^2^ = 62.311, *df* = 2, *P*<0.0001). The IR *D*. *albomicans* strain was more suited as a host, with wasp emergence of 44–68% compared to the NH (51–54.3%) and TP (35.2–40.2%) strains (Fisher’s: IR—NH, IR-TP: *P*<0.0001). Fly emergence was particularly low in the IR strain, below 3%.

### Host Choice Experiments

**Fig 6:**
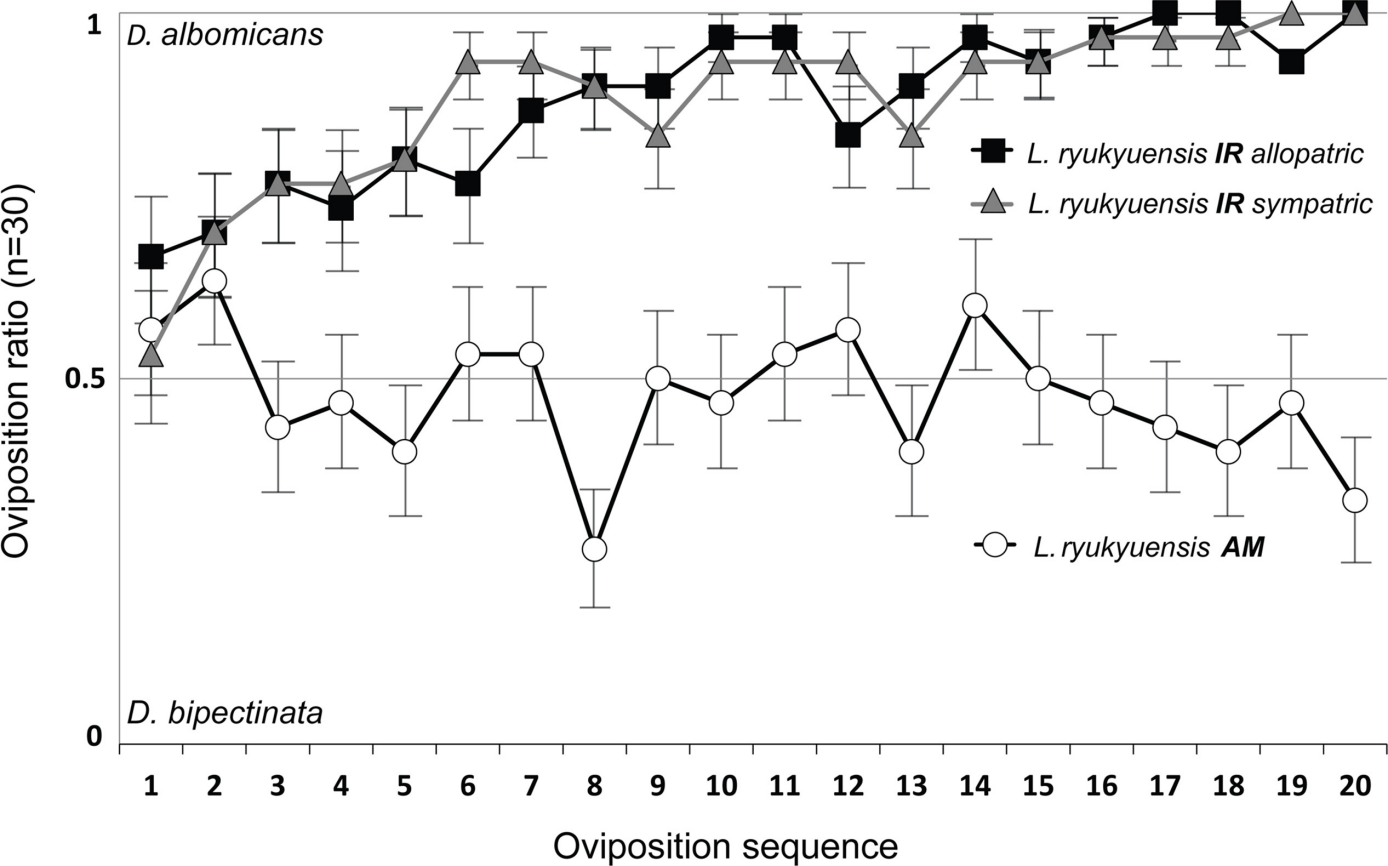
Oviposition sequences of AM and IR strains of *L*. *ryukyuensis*. Marks indicate the departure from the 0.5 line which represents the same probability of *D*. *albomicans* or *D*. *bipectinata* larvae being chosen for oviposition. Each mark represents the ratio derived from the observation of 30 wasps. Numbers 1 and 20 on the horizontal axis represent the first and the last observed oviposition, respectively. (white circles) AM wasps ovipositing in NH fly strains, (black squares) IR wasps ovipositing in NH fly strains, (gray triangles) IR wasps ovipositing in IR fly strains.

**Fig 7:**
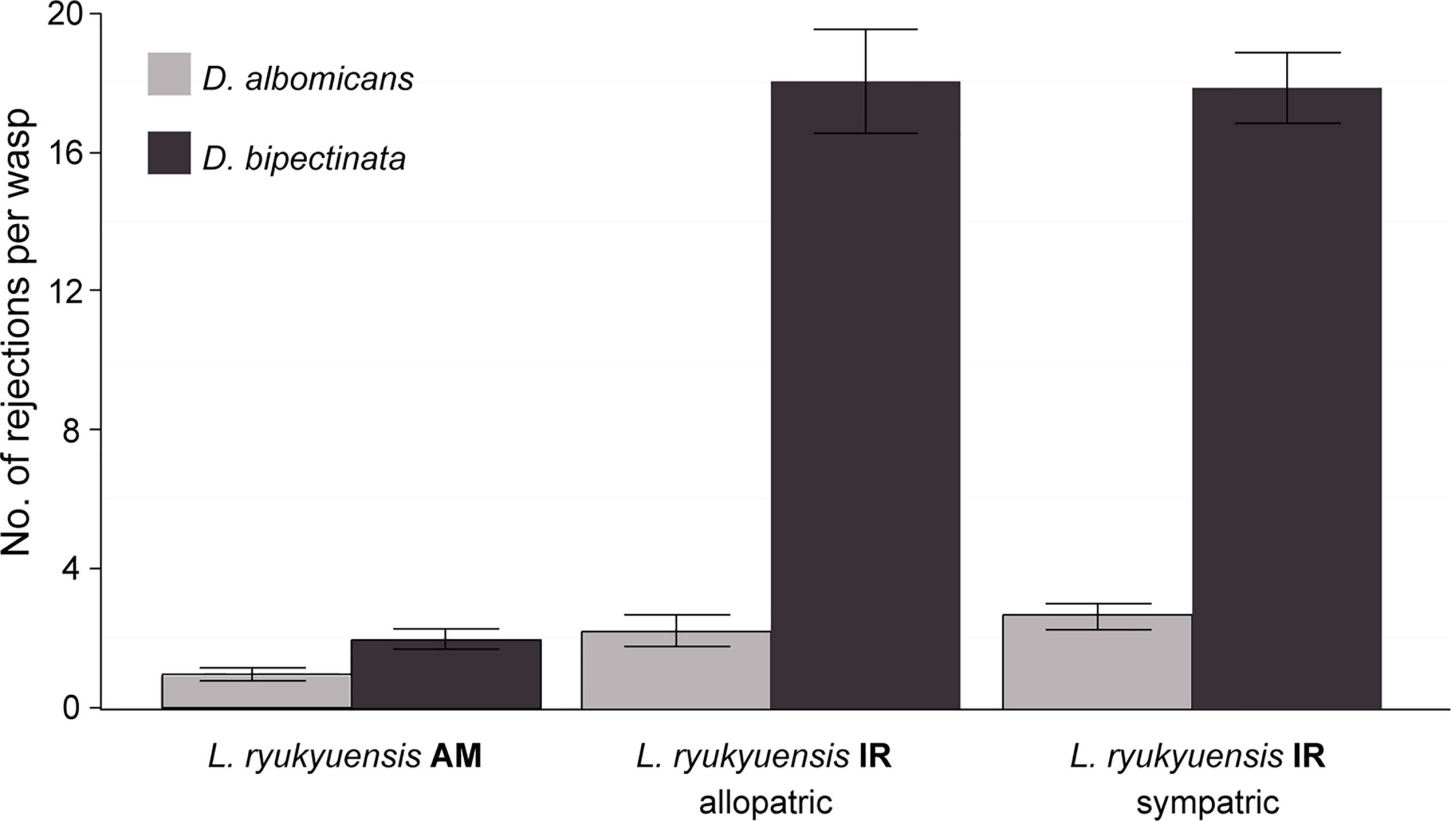
Number of rejections per wasp during an oviposition sequence. Rejection of NH flies by AM wasps, NH flies by IR wasps, and IR flies by IR wasps. Oviposition sequence is measured as 20 successful subsequent oviposition events. Average and standard error are shown.

There were marked differences in the oviposition behavior of AM and IR wasp strains (2-sample test for equality of proportions: χ^2^ = 223.061, *df* = 1, *P*<0.0001). While the oviposition of the AM wasps did not significantly deviate from a random pattern, with equal probabilities of oviposition in either fly species, the IR wasps clearly preferred to oviposit in *D*. *albomicans* (Binomial: *P*<0.0001) (Fig 6). We observed a trend where the probability that IR wasps would oviposit in *D*. *albomicans* increased with experience. Furthermore, IR wasps rejected an average of 20.28 ± 3.60 larvae per observed oviposition sequence, markedly more compared to AM wasps, with 2.9 ± 0.78 rejected larvae on average (Fig 7). Finally, *D*. *bipectinata* larvae were more rejected by IR wasps (18.04 ± 3.1) than *D*. *albomicans* larvae (2.24 ± 0.91).

Alternate coloring of host larvae had no impact on the results in experiments with IR wasps, but may have affected the AM wasp choice when *D*. *albomicans* was colored red (*P*<0.05). Nonetheless, unlike the trials with IR wasps, we observed no trend in relation to wasp experience. No differences were observed between the oviposition sequences of IR wasps in sympatric (IR) and allopatric (NH) fly strains (S2 Supporting Information). Eggs were present in over 98% of larvae dissected after the experiment.

## Discussion

All four species had species-specific phylogeographic patterns, with no apparent correlation between the patterns of the parasitoid and any of the *Drosophila* species. *D*. *albomicans* had the highest diversity among the three fly species for both COI and *Gpdh*. The diversity of COI in this species was extremely high, with 48 haplotypes recovered from 101 adult flies. This finding is in accordance with the results of Chen *et al*. [46] and Wang *et al*. [47], who report remarkable polymorphism in *D*. *albomicans* populations. Wang *et al*. [47] suggest that a mechanism which maintains mtDNA diversity exists in this fly. *D*. *albomicans* was also the only species where we observed evidence of restricted gene flow. Based on COI markers, there is divergence between the northern (KG, AM, NH) and the southern (IR, TP) population group of this species. The number of populations we used in the AMOVA can produce a minimum *P*-value of 0.1, which in this case does not allow us to quantitatively reject or accept the null hypothesis of no group structure [48]. Nonetheless, 32.3% of total variation was attributed to the variation between the northern and the southern population-group, and all pairwise *F*_ST_ comparisons indicated significant between-group differentiation. No significant relationship was found between genetic divergence and geographic distance in *D*. *albomicans*, suggesting that rather than simple isolation by distance, the Kerama strait (>250 km) in the island chain represents a strong barrier for gene flow in this particular species. This is further supported by the two separate expansion demographic events, estimated at c.724 ka (497–866 ka) for the southern, and c. 122 ka (15–242 ka) for the northern populations. According to the Kizaki and Oshiro`s hypothesis modified by Hikida and Ota [12], and Osozawa *et al*. [49], the Kerama gap was already wide in the middle Pleistocene, which corresponds to the first expansion of the southern populations, and may explain why the southern haplotypes did not easily spread further north. Possible land bridges over the gap [50] may have aided expansion to the north in a single or several occasions, but these were probably sparse or followed by severe bottlenecks, and most likely one-way (south to north) events. The Tokara gap does not seem to be a barrier for this species, as the northern populations are widespread up to Kagoshima (KG). *Gpdh* of *D*. *albomicans* had lower haplotype diversity and little geographical structure. COI should, in theory, show more structure than typical single-copy nuclear DNA, because of its faster mutation rate, smaller effective population size, and larger susceptibility to genetic drift [51, 52]. There is also the possibility of male-biased dispersal. In species in which females are philopatric and males disperse, the uni-parentally inherited COI markers are expected to show more genetic differentiation than bi-parentally inherited nuclear markers [53]. Male—biased dispersal has been previously reported in *Drosophila*, in the instance of *D*. *pachea* [54].

*Drosophila takahashii* had a star-like COI haplotype network, with an expansion event estimated at c. 196 ka (151–255 ka). This timeframe roughly corresponds to the expansion time estimated for the northern populations of *D*. *albomicans*, indicating favorable climate and/or geography-related factors in that particular period. This period additionally corresponds to the occurrence of land-bridges over both Tokara and Kerama gaps according to Kimura [50]. The lowest nuclear *Gpdh* diversity among the three fly species further suggests that *D*. *takahashii* may have expanded more recently than the other two fly species, and even more recently than *L*. *ryukyuensis*.

Both *D*. *bipectinata* and *L*. *ryukyuensis* had very low COI marker diversity. This kind of extremely low mitochondrial diversity may result from a selective sweep, potentially due to a *Wolbachia* infection [27]. Ravikumar *et al*. [55] found that *D*. *bipectinata* from India was infected by *Wolbachia* supergroup A, subgroup Mel, whereas Indian *D*. *albomicans* and *D*. *takahashii* were *Wolbachia* free. Prompted by discrepancies in mitochondrial and nuclear diversity of *D*. *bipectinata* and *L*. *ryukyuensis*, we attempted to amplify *Wolbachia* sequences from all four species using conserved primers against the *Wolbachia* surface protein gene (*wsp*) and the filamenting temperature sensitive gene Z (*ftsZ*) (S1 Supporting Information), but were not successful. Further analyses are needed to shed some light on whether the *Wolbachia* infection has indeed spread in *D*. *bipectinata* as far as the Ryukyu archipelago and Taiwan, or whether it has affected *L*. *ryukyuensis* populations.

Why does *D*. *albomicans* show population differentiation and the other species do not? Additionally, why does the Kerama gap represent a barrier for the dispersal of *D*. *albomicans*, and not for *D*. *takahashii*, nor for *D*. *bipectinata*? Firstly, the likely scenario is that these three species have colonized these islands at different points in time, and that a longer history in these islands would result in more diversity and more differentiation between the islands in the presence of barriers. Secondly, it is possible that different dispersal abilities, feeding, and/or breeding preferences of these species influence the observed phylogeographic patterns. A similar case was observed in the populations of three sympatric cactophilic *Drosophila* from Sonoran Desert, where the Sea of Cortez was an effective dispersal barrier for only one of the three studied species, despite them having similar niches and overlapping distributions [56]. Our three fly species are all frugivorous, but may prefer different types of habitat. In banana-baited traps 87% of *D*. *albomicans* was collected from the forest, while over 50% of *D*. *takahashii* and *D*. *bipectinata* were obtained from open lands and domestic areas [22]. These differences may translate into easier dispersal, or higher responsiveness to corridors, especially ones with scarcer vegetation such as potential land-bridges. Smaller body size of *D*. *takahashii* and *D*. *bipectinata* compared to *D*. *albomicans* may additionally be of advantage in wind-aided dispersal. We believe the observed phylogeographic differences between our fly species may result from a combination of both of these factors—temporal and ecological, but further studies are needed to shed more light on dispersal abilities and corridor responsiveness in these species.

Based on the low diversity and inter-island differentiation in COI and ITS2 sequences, we conclude that the parasitoid *L*. *ryukyuensis* disperses relatively well. Higher ITS2 diversity in the south (IR, TP) suggests a northward expansion. There was no evidence for a tight coevolutionary interaction between the parasitoid and any of the fly species, based on phylogeographic patterns. Instead, molecular marker results are more in agreement with a scenario where parasitoids alternate between hosts.

Looser coevolutionary interactions, where a parasitoid attacks several host species, are more likely to lead to differentiation in interaction traits between islands. In our study, the northern AM *L*. *ryukyuensis* strain was more likely to accept as a host and oviposit in less suitable *D*. *takahashii*, or non-suitable *D*. *bipectinata*, while the southern IR wasp strain was least likely to accept them. Differences in host suitability were observed in *D*. *albomicans*, a current major host for this wasp species. The IR strain of *D*. *albomicans* was the least resistant and, therefore, more suited as a host for *L*. *ryukyuensis* compared to the NH and TP strains. Phylogeographic structure in this species indicates that there is restricted gene flow between the island populations that could facilitate differentiation in defense traits related to host suitability.

Host-choice experiments confirmed that the IR wasp strain clearly preferred *D*. *albomicans* to *D*. *bipectinata*. After experiencing oviposition in both potential host species, wasps would reject *D*. *bipectinata* larvae in favor of *D*. *albomicans*. The results are consistent with host-acceptance experiments, where IR wasps oviposited less in *D*. *bipectinata* even when no other host was available. The AM wasp strain oviposited equally well in both species, both in no-choice and choice assays. We can think of two reasons for the observed differences. If we take into the account that based on phylogeographic patterns, our species expanded from south to north, then the southern populations (IR) would have had more time to adapt by rejecting hosts they develop less successfully in. On the other hand, the northern *L*. *ryukyuensis* populations (AM) are on the `frontier of expansion`. Geographic edges of species ranges, such as AM strains for *L*. *ryukyuensis* and *D*. *takahashii*, where significant differences in host-acceptance were also observed, are especially likely to be highly dynamic zones for the evolution of new traits, as the species reach regions with different abiotic conditions and/or community composition [19]. Parasitoids, in this case, may benefit from accepting a broader host spectrum, enabling them to colonize new areas.

There is valuable information to be gained by focusing on a broader spectrum of abundant potential host species, other than the obvious host in the field *D*. *albomicans*. Despite generally not recognizing or accepting *D*. *takahashii* as a host, *L*. *ryukyuensis* can successfully develop in this species. This interaction is further more likely to occur in northern populations, located on the edge of both species’ ranges. On the other hand, *D*. *bipectinata* is readily oviposited in, especially in northern populations where the wasps do not distinguish between *D*. *bipectinata* and the better host *D*. *albomicans*. Conversely, *D*. *bipectinata* is almost completely resistant to *L*. *ryukyuensis*. *D*. *bipectinata* may have been a former host of *L*. *ryukyuensis*, which could explain both the defense against this wasp, and the residual recognition and acceptance of *D*. *bipectinata* as a host. Another possibility is that the defense of *D*. *bipectinata* was developed in response to another parasitoid, or is a general defensive response to multiple parasitoids. Based on our results, *D*. *bipectinata* may be actively oviposited in by *L*. *ryukyuensis* across the region, in which case this wasp would still be exerting a selective pressure on the maintenance of defense mechanisms in this fly species. We hope to further explore this subject in future surveys.

In our study there was differentiation in both molecular markers and interaction traits in *D*. *albomicans*. However, inter-population differences in host-acceptance traits, in both *D*. *takahashii* and *L*. *ryukyuensis*, and the behavioral differences in *L*. *ryukyuensis*, evolved despite there being little evidence of molecular differentiation. This discrepancy is most likely due to the use of neutral markers. Phylogeographic data in this case underestimates the amount of differentiation in traits that are under selection in interspecific interactions [19]. It is possible that host-acceptance related traits evolve faster compared to defense traits. Both incongruent phylogeographic patterns and differentiation in interaction patterns between the islands point to a dynamic coevolutionary process, with different evolutionary trajectories in different island populations.

## Supporting Information

S1 Fig Pairwise mismatch distribution for the northern and the southern population-group of *D*. *albomicans* based on partial COI sequences. (TIF)

S2 Fig Correlation analyses of inter-population genetic distance and geographic distance between pairwise populations. (TIF)

S1 Supporting Information DNA extraction protocol, PCR reaction protocol and the list of primers used for amplification of COI, ITS2 and amplification and disambiguation of *Gpdh*. (PDF)

S2 Supporting Information Host choice experiment results. (PDF)

S1 Table Host acceptance experiment results. (PDF)

S2 Table Host suitability experiment results. (PDF)
