# Supplementary figures and images for: Phylogeography, Interaction Patterns and the Evolution of Host Choice in Drosophila-Parasitoid Systems in Ryukyu Archipelago and Taiwan
Source: PLoS One. 2015 Jun 12;10(6):e0129132. doi: 10.1371/journal.pone.0129132 (PMC4466491; doi:10.1371/journal.pone.0129132)

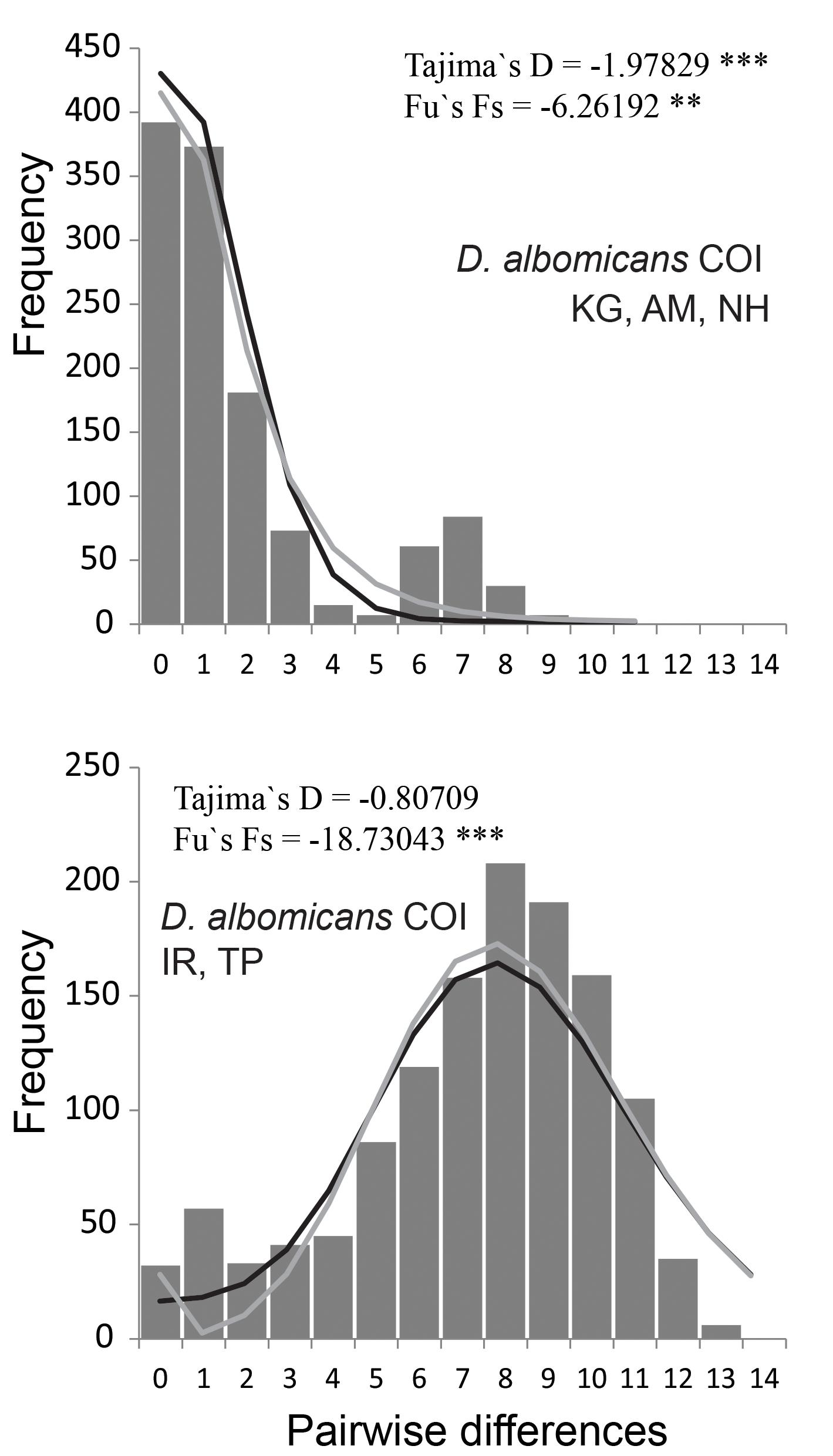

Supplement: S1 Fig — (TIF) [file pone.0129132.s001.tif]

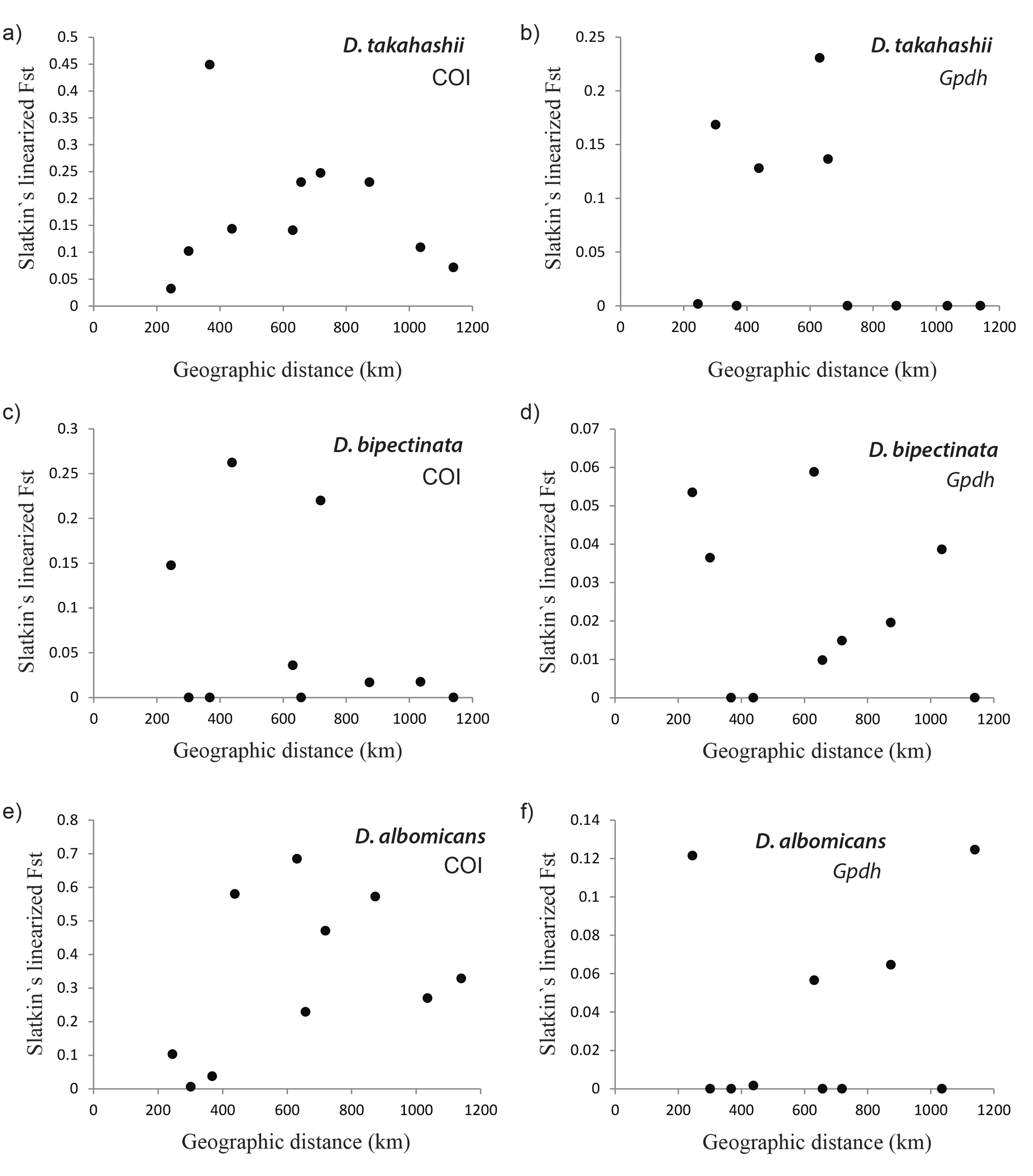

Supplement: S2 Fig — (TIF) [file pone.0129132.s002.tif]
