# Supplementary material for: Phylogeography, Interaction Patterns and the Evolution of Host Choice in Drosophila-Parasitoid Systems in Ryukyu Archipelago and Taiwan
Source: PLoS One. 2015 Jun 12;10(6):e0129132. doi: 10.1371/journal.pone.0129132 (PMC4466491; doi:10.1371/journal.pone.0129132)
Supplement: S1 Supporting Information — (PDF) [file pone.0129132.s003.pdf]

DNA Isolation Protocol:

1. Samples were homogenized in 500 µl extraction buffer (0.5% SDS, 10mM EDTA, 10mM Tris-HCl, pH 8.0) and 12.5 µl of Proteinase K was added.
2. Samples were incubate with shaking at 56 ˚C for 1 h, and then at 37 ˚C overnight.
3. After adding 500 µl of phenol to each tube, samples were incubated at room temperature with shaking for over 6 h.
4. Samples were centrifuged for 5 minutes at 13,000 rpm – or longer until the two phases clearly separated.
5. The upper aqueous phase was transferred into a fresh tube, and 500 µl of phenol-chloroform (1:1) was added to each sample. Samples were incubated with shaking for over 6 h.
6. Samples were centrifuged for 5 minutes at 13,000 rpm.
7. Upper phase was transferred into a fresh tube, and 500 µl of chloroform was added to each sample. Samples were incubated with shaking at room temperature for over 6 h.
8. Samples were centrifuged for 5 minutes at 13,000 rpm.
9. The upper phase was transferred into a new tube, 1000 µl of 100 % ethanol was added, and each tube was vortexed and incubated with shaking for 1h.
10. Samples were centrifuged for 15 minutes at 13,000 rpm.
11. Liquid phase was removed, 500 µl of 70 % ethanol was added and each tube was vortexed.
12. Samples were centrifuged for 5 minutes at 13,000 rpm.
13. Ethanol was removed, tubes were dried for 10-15 minutes, and an arbitrary volume of TE buffer was added (100 – 200 µl) based on the amount of DNA extracted.

PCR profile:

17.08 µl	H_2O
2.5 µl	10x buffer
1.24 µl	primer 1 (100 µM stock)
1.24 µl	primer 2 (100 µM stock)
0.5 µl	dNTP
0.2 µl	Taq Polymerase
1 µl	DNA sample

94˚C 10 min #denaturation

30 cycles of:

- **94˚C** 1 min #denaturation
- **50˚C** 1 min #annealing
- **72˚C** 1.5 min #extension

72˚C 10 min #final extension

Primers used for the amplification of COI, amplification and disambiguation of *Gpdh*, amplification of ITS2, *wsp* and *ftsZ*

**COI**	
LCO	5'-GGTCAACAAATCATAAAGATATTGG-3' [1]
HCO	5'-TAAACTTCAGGGTGACCAAAAAATCA-3' [1]
Gpdh	
GNL-mel	5'-GTGGTGCCCCACCAGTTCAT-3' [2]
GNR-mel	5'-GGCTTGAGCTGATTTGTGCA-3' [2]
L4BN	5'-CCATGYGCTGTCTTGATGGG-3' [3]
R4M	5'-ACAGCCGCCTTGGTGTTGTCGCC-3' [3]
bipecti2a	5'-ACGATGTTCTTGAGGGCAA-3'
bipecti2b	5'-ACGATGTTCTTGAGGGCAG-3'
bipecti3a	5'-GCATATTATCACGCGACAT-3'
bipecti3b	5'-GCATATTATCACGCGACAC-3'
bipecti320C	5'-GTTCTTGAGGGCACCACACACCTCG-3'
bipecti320G	5'-GTTCTTGAGGGCACCACACACCTCC-3'
bipecti209C	5'-CAACGAAGTGGCTGAGGGCAAC-3'
bipecti209T	5'-CAACGAAGTGGCTGAGGGCAAT-3'
bipecti209A	5'-CAGCCGATGGTTGTCTCGCARAAA-3'
bipecti209G	5'-CAGCCGATGGTTGTCTCGCARAAG-3'
takaF	5'-TCAAGGGTTTCGACAAGGCCGA-3'
takaR	5'-CAGCTCTCGAAGAAGGTGGACA-3'
albo109A	5'-AGGTGAGTCAATGGACGTTCTTTCA-3'
albo109G	5'-AGGTGAGTCAATGGACGTTCTTTCG-3'
albo128A	5'-CTTTCGTAGTATATTTAGAACACAA-3'
albo128C	5'-CTTTCGTAGTATATTTAGAACACAC-3'
albo162A	5'-CTTTGAAAACCCCTTTGCTAATCTA-3'
albo162C	5'-CTTTGAAAACCCCTTTGCTAATCTC-3'
albo162G	5'-CTTTGAAAACCCCTTTGCTAATCTG-3'
albo162r_C	5'-TATCTGAGCGTGGGAGGAGCGTGTC-3'
albo162r_G	5'-TATCTGAGCGTGGGAGGAGCGTGTG-3'
albo162r_T	5'-TATCTGAGCGTGGGAGGAGCGTGTT-3'

**ITS2**	
f	5'-TGTCAACTGCAGGACACATG-3' [4]
r	5'-AATGCTTAAATTTAGGGGGTA-3' [4]
wsp	
wsp1	5'-GGATCCGGGTCCAATAAGTGATGAAGAAAC-3′ [5]
wsp2	5'-GGATCCTTAAAACGCTACTCCAGCTTCTGC-3′ [5]
ftsZ	
fts1	5'-GTATGCCGATTGCAGAGCTTG-3′ [6]
fts2	5'-GCCATGAGTATTCACTTGGCT-3′ [6]

[1] Folmer O, Black M, Hoeh W, Lutz R, Vrijenhoek R. DNA primers for amplification of mitochondrial cytochrome c oxidase subunit I from diverse metazoan invertebrates. Mol Mar Biol Biotechnol. 1994; 3: 294–299.

[2] Kopp A, Barmina O. Evolutionary history of the *Drosophila bipectinata* species complex. Genet Res. 2005; 85: 23-46.

[3] Barrio E, Ayala FJ. Evolution of the *Drosophila obscura* species group inferred from the Gpdh and Sod genes. Molec Phylog Evol. 1997; 7: 79—93.

[4] Campbell BC, Steffen-Campbell JD, Werren JH. Phylogeny of the *Nasonia* species complex (Hymenoptera: Pteromalidae) inferred from an internal transcribed spacer (ITS2) and 28S rDNA sequences. Insect Mol Biol. 1993; 2:225-37.

[5] Kondo N, Shimada M, Fukatsu T. High Prevalence of *Wolbachia* in the Azuki Bean Beetle *Callosobruchus chinensis* (Coleoptera, Bruchidae). Zool Sci. 1999; 16:955-962.

[6] Holden P R, Brookfield JFY, Jones P. Cloning and characterization of an ftsZ homologue from a bacterial symbiont of *Drosophila melanogaster*. Mol Gen Genet 1993; 240:213–220.
