# Supplementary material for: Phylogeography, Interaction Patterns and the Evolution of Host Choice in Drosophila-Parasitoid Systems in Ryukyu Archipelago and Taiwan
Source: PLoS One. 2015 Jun 12;10(6):e0129132. doi: 10.1371/journal.pone.0129132 (PMC4466491; doi:10.1371/journal.pone.0129132)
Supplement: S2 Supporting Information — (PDF) [file pone.0129132.s004.pdf]

L. ryukyuensis **AM,** ***D. albomicans*** **NH (1 - green),** ***D. bipectinata*** **NH (0 - red)**

		1	2	3	4	5	6	7	8	9	10	11	12	13	14	15	16	17	18	19	20	reject ALBO	reject BIP
D. bipectinata (colored)	I	0	1	1	1	1	0	0	0	1	0	0	0	0	1	0	1	1	0	0	0	0	0
	II	1	1	0	1	0	0	1	0	1	1	1	1	0	0	1	1	1	1	0	1	1	0
	III	0	0	0	1	0	1	1	1	1	0	0	1	1	1	1	1	1	1	0	0	1	0
	IV	0	1	1	0	0	1	0	1	1	1	0	1	1	1	1	0	1	1	1	1	0	0
	V	1	0	1	1	0	1	1	1	0	0	1	1	0	1	1	1	0	1	1	1	1	1
	VI	1	1	1	1	0	0	1	1	0	0	1	0	1	0	0	1	1	0	1	0	0	2
	VII	0	0	1	1	1	1	1	0	1	0	1	1	1	1	1	1	1	0	0	0	0	1
	VIII	1	1	0	1	1	1	0	1	1	1	0	1	1	0	0	1	0	0	0	0	1	4
	IX	1	1	0	0	0	0	0	0	0	1	0	0	1	1	0	0	0	1	0	0	2	1
	X	0	1	0	1	1	1	1	0	1	0	1	0	0	0	1	0	1	0	0	1	1	4
	XI	0	0	1	1	1	1	1	0	0	0	1	1	1	1	0	1	0	0	0	0	1	4
	XII	1	1	0	0	1	1	0	0	0	0	1	1	0	0	1	0	0	0	1	0	0	3
	XIII	0	0	1	0	0	0	1	1	1	0	0	0	0	1	0	0	0	1	0	0	2	5
	XIV	0	0	1	0	1	0	0	0	1	1	1	0	0	1	0	0	1	1	1	0	2	4
	XV	1	1	0	1	0	1	0	1	0	1	1	1	0	0	1	1	0	0	1	0	1	2
D. albomicans (colored)	I	0	1	0	0	0	1	0	0	1	1	1	0	1	0	1	1	1	0	0	0	0	2
	II	0	0	1	1	1	1	0	0	0	0	1	1	0	1	1	0	0	0	0	0	0	3
	III	1	0	0	1	0	1	0	0	1	0	1	1	1	0	0	1	0	0	0	0	1	0
	IV	1	1	0	0	0	0	0	0	0	1	0	0	0	1	0	0	0	0	0	0	0	0
	V	1	1	0	0	0	0	1	0	0	0	0	0	0	0	1	0	0	0	0	0	0	1
	VI	1	0	0	0	1	0	0	0	1	1	0	1	0	1	1	0	0	0	1	1	3	5
	VII	0	1	0	0	0	0	1	0	0	0	1	1	1	0	0	0	1	0	1	0	1	1
	VIII	1	1	0	0	0	0	1	0	0	0	0	0	0	0	1	1	0	1	1	0	1	2
	IX	0	1	0	0	0	1	0	0	1	0	1	1	0	1	0	0	0	1	1	1	1	3
	X	1	1	0	0	0	0	1	0	0	1	0	1	1	1	0	0	1	1	0	1	1	2
	XI	1	1	0	0	1	1	1	0	0	0	1	0	0	1	1	0	0	1	1	0	3	2
	XII	1	0	1	1	1	0	1	0	1	1	0	0	1	1	0	1	1	0	1	1	1	1
	XIII	0	1	1	0	0	1	1	1	0	1	0	1	0	1	1	0	0	1	1	1	1	2
	XIV	1	1	1	1	0	1	0	0	0	1	0	1	0	0	0	0	1	0	1	1	3	3
	XV	1	0	1	0	1	0	1	0	1	1	1	0	0	1	0	1	0	0	0	0	0	0

***L. ryukyuensis* IR, *D. albomicans* NH (1 - green), *D. bipectinata* NH (0 - red)**

Group		1	2	3	4	5	6	7	8	9	10	11	12	13	14	15	16	17	18	19	20	reject ALBO	reject BIP
D. bipectinata (colored)	I	1	1	1	1	1	1	1	1	1	1	1	1	1	1	1	1	1	1	1	1	6	24
	II	1	1	1	1	0	1	1	1	1	1	1	1	1	1	1	1	1	1	1	1	7	21
	III	1	0	1	1	1	1	1	1	1	1	1	1	1	1	1	1	1	1	1	1	5	14
	IV	1	1	1	1	1	1	0	1	1	1	1	0	1	1	1	1	1	1	0	1	2	5
	V	1	1	0	0	1	1	1	1	1	1	0	1	1	1	1	1	1	1	1	1	3	23
	VI	1	1	1	0	1	1	1	1	1	1	1	0	1	1	1	1	1	1	1	1	3	8
	VII	1	1	0	0	1	1	1	0	1	1	1	0	1	1	1	0	1	1	1	1	0	6
	VIII	0	0	1	0	0	0	0	1	1	0	1	1	1	1	1	1	1	1	1	1	0	12
	IX	0	1	1	1	1	1	1	1	1	1	1	1	1	1	1	1	1	1	1	1	7	30
	X	1	1	0	1	1	1	0	1	1	1	1	1	1	1	1	1	1	1	1	1	3	20
	XI	0	1	1	1	1	1	1	1	1	1	1	1	0	1	1	1	1	1	1	1	1	27
	XII	1	1	0	1	1	1	1	1	1	1	1	1	1	1	1	1	1	1	1	1	2	19
	XIII	1	0	1	1	1	0	1	0	0	1	1	0	1	1	1	1	1	1	1	1	1	13
	XIV	0	0	0	1	1	1	1	1	1	1	1	1	1	1	1	1	1	1	1	1	n.a.	n.a.
	XV	0	1	0	1	1	0	1	1	1	1	1	1	1	1	1	1	1	1	1	1	n.a.	n.a.
D. albomicans (colored)	I	1	1	1	1	1	0	1	1	1	1	1	1	1	1	1	1	1	1	1	1	5	30
	II	1	0	1	1	1	0	1	0	1	1	1	1	1	1	0	1	1	1	1	1	1	17
	III	0	0	1	0	0	1	1	1	0	1	1	1	1	0	1	1	1	1	1	1	2	16
	IV	1	1	1	1	0	0	1	1	1	1	1	1	0	1	1	1	1	1	1	1	0	11
	V	0	1	1	1	1	1	1	1	1	1	1	1	1	1	1	1	1	1	1	1	3	21
	VI	1	1	1	0	0	1	1	1	1	1	1	0	1	1	1	1	1	1	1	1	0	21
	VII	0	1	0	1	1	1	1	1	1	1	1	1	1	1	1	1	1	1	1	1	0	19
	VIII	1	0	1	1	0	0	1	1	1	1	1	1	1	1	1	1	1	1	1	1	0	4
	IX	0	1	1	1	1	1	1	1	1	1	1	1	1	1	1	1	1	1	1	1	1	27
	X	1	1	1	0	1	1	0	1	1	1	1	1	0	1	0	1	1	1	1	1	1	17
	XI	1	1	1	0	1	1	1	1	1	1	1	1	1	1	1	1	1	1	1	1	2	26
	XII	0	1	1	1	1	1	1	1	1	1	1	1	1	1	1	1	1	1	1	1	1	20
	XIII	1	1	1	1	1	1	1	1	0	1	1	1	1	1	1	1	1	1	0	1	n.a.	n.a.
	XIV	1	0	1	1	1	1	1	1	1	1	1	1	1	1	1	1	1	1	1	1	n.a.	n.a.
	XV	1	0	1	1	1	1	1	1	1	1	1	1	1	1	1	1	1	1	1	1	n.a.	n.a.

L. ryukyuensis IR, _D. albomicans_ IR (1 - green), _D. bipectinata_ IR (0 - red)

Group		1	2	3	4	5	6	7	8	9	10	11	12	13	14	15	16	17	18	19	20	reject ALBO	reject BIP
D. bipectinata (colored)	I	0	1	1	1	1	1	1	0	1	1	1	1	1	1	1	1	1	1	1	1	4	20
	II	1	1	1	0	1	1	0	1	1	1	1	1	0	1	1	1	1	1	1	1	2	9
	III	0	1	0	1	0	0	1	0	1	1	1	1	0	1	1	1	1	1	1	1	4	23
	IV	0	0	1	1	1	1	1	1	1	1	1	1	1	1	1	1	1	1	1	1	1	13
	V	1	0	0	0	1	0	1	1	0	1	0	1	1	1	1	1	0	1	1	1	0	7
	VI	0	1	1	1	1	1	1	1	1	1	1	1	1	1	1	1	1	1	1	1	6	26
	VII	0	1	0	1	1	1	1	1	1	1	1	1	1	1	1	1	1	1	1	1	7	23
	VIII	0	1	1	1	1	1	1	1	0	1	1	1	1	1	1	1	1	1	1	1	1	12
	IX	1	1	1	1	1	1	1	1	1	1	1	1	1	1	1	1	1	1	1	1	5	14
	X	1	0	1	1	1	1	1	1	1	1	1	1	1	1	1	1	1	1	1	1	3	22
	XI	0	1	1	1	1	1	1	1	1	1	1	1	1	1	1	1	1	1	1	1	3	24
	XII	1	1	1	1	1	1	1	1	0	1	1	1	1	1	0	1	1	1	1	1	2	14
	XIII	0	1	1	1	0	1	1	1	1	1	1	1	0	1	1	1	1	1	1	1	2	10
	XIV	1	0	1	1	1	1	1	1	1	1	1	1	1	1	0	1	1	1	1	1	3	12
	XV	1	1	1	0	1	1	1	1	1	1	1	1	1	1	1	1	1	1	1	1	1	18
D. albomicans (colored)	I	0	0	1	1	0	1	0	1	1	1	1	1	1	0	1	0	1	0	1	1	1	12
	II	0	1	0	1	0	1	1	1	0	0	1	1	1	1	1	1	1	1	1	1	1	22
	III	0	1	1	1	1	1	1	1	0	1	1	1	1	1	1	1	1	1	1	1	4	21
	IV	1	1	1	1	0	1	1	1	1	1	1	1	1	1	1	1	1	1	1	1	1	26
	V	0	1	1	0	1	1	1	1	1	1	1	1	0	1	1	1	1	1	1	1	1	18
	VI	0	0	0	1	1	1	1	1	1	1	1	1	1	1	1	1	1	1	1	1	2	22
	VII	1	1	1	1	1	1	1	1	1	1	1	1	1	1	1	1	1	1	1	1	3	20
	VIII	1	0	0	1	1	1	1	1	1	1	1	0	1	1	1	1	1	1	1	1	1	18
	IX	1	0	0	0	1	1	1	1	1	0	1	0	1	0	1	1	1	1	1	1	2	15
	X	1	1	1	0	0	1	1	1	1	1	0	1	1	1	1	1	1	1	1	1	1	21
	XI	1	1	1	1	1	1	1	0	1	1	1	1	1	1	1	1	1	1	1	1	3	12
	XII	1	1	1	1	1	1	1	1	1	1	1	1	1	1	1	1	1	1	1	1	2	18
	XIII	1	1	1	0	1	1	1	1	1	1	1	1	1	1	1	1	1	1	1	1	10	16
	XIV	0	1	1	1	1	1	1	1	1	1	1	1	0	1	1	1	1	1	1	1	3	30
	XV	1	0	1	1	1	1	1	1	1	1	1	1	1	1	1	1	1	1	1	1	2	19
