# Supplementary material for: Phylogeography, Interaction Patterns and the Evolution of Host Choice in Drosophila-Parasitoid Systems in Ryukyu Archipelago and Taiwan
Source: PLoS One. 2015 Jun 12;10(6):e0129132. doi: 10.1371/journal.pone.0129132 (PMC4466491; doi:10.1371/journal.pone.0129132)
Supplement: S1 Table — (PDF) [file pone.0129132.s005.pdf]

		D. albomicans				*D. takahashii*			*D. bipectinata*			
L. ryukyuensis		**AM**	**NH**	**IR**	**TP**	**AM**	**NH**	**IR**	**AM**	**NH**	**IR**	**TP**
	**N**	4	112	106	110	115	108	110	106	110	100	109
**AM**	**eggs**	3	108	98	101	47	24	17	96	98	97	95
	**%**	75	96.42857	92.45283	91.81818	40.86957	22.22222	15.45455	90.56604	89.09091	97	87.15596
	**N**	0	104	123	108	105	104	107	106	105	102	102
**NH**	**eggs**	0	100	113	103	21	11	8	70	88	91	92
	**%**	0	96.15385	91.86992	95.37037	20	10.57692	7.476636	66.03774	83.80952	89.21569	90.19608
	**N**	10	118	115	102	120	102	104	106	105	100	106
**IR**	**eggs**	9	108	107	92	11	1	10	50	42	68	60
	**%**	90	91.52542	93.04348	90.19608	9.166667	0.980392	9.615385	47.16981	40	68	56.60377
