# Supplementary material for: Phylogeography, Interaction Patterns and the Evolution of Host Choice in Drosophila-Parasitoid Systems in Ryukyu Archipelago and Taiwan
Source: PLoS One. 2015 Jun 12;10(6):e0129132. doi: 10.1371/journal.pone.0129132 (PMC4466491; doi:10.1371/journal.pone.0129132)
Supplement: S2 Table — (PDF) [file pone.0129132.s006.pdf]

D. takahashii	*L. ryukyuensis*	Fly	Wasp	Total
	**AM**	70	13	103
**AM**	**NH**	90	2	110
	**IR**	81	2	100
	**AM**	96	1	120
**NH**	**NH**	90	2	105
	**IR**	86	0	102
	**AM**	63	4	100
**IR**	**NH**	58	4	102
	**IR**	68	0	108
D. bipectinata	***L. ryukyuensis***	**Fly**	**Wasp**	**Total**
	**AM**	85	0	106
**AM**	**NH**	81	0	114
	**IR**	105	0	115
	**AM**	115	0	120
**NH**	**NH**	95	0	114
	**IR**	101	0	121
	**AM**	100	0	111
**IR**	**NH**	91	0	103
	**IR**	72	1	100
	**AM**	88	0	121
**TP**	**NH**	106	0	130
	**IR**	91	0	120
D. albomicans	***L. ryukyuensis***	**Fly**	**Wasp**	**Total**
	**AM**	23	57	105
**NH**	**NH**	15	51	100
	**IR**	19	54	105
	**AM**	3	68	100
**IR**	**NH**	3	45	102
	**IR**	0	63	101
	**AM**	19	37	101
**TP**	**NH**	24	41	102
	**IR**	25	38	108
